# Parameter Optimization in High-Throughput Testing for Structural Materials

**DOI:** 10.3390/ma12203439

**Published:** 2019-10-21

**Authors:** Alexander Bader, Anastasiya Toenjes, Nicole Wielki, Andreas Mändle, Ann-Kathrin Onken, Axel von Hehl, Daniel Meyer, Werner Brannath, Kirsten Tracht

**Affiliations:** 1 Bremen Institute for Mechanical Engineering (bime), University of Bremen, Badgasteiner Str. 1, 28359 Bremen, Germany; onken@bime.de (A.-K.O.); tracht@bime.de (K.T.); 2 Leibniz Institute for Materials Engineering—IWT, University of Bremen, Badgasteiner Str. 3, 28359 Bremen, Germany; toenjes@iwt-bremen.de (A.T.); wielki@iwt-bremen.de (N.W.); vonhehl@iwt-bremen.de (A.v.H.); dmeyer@iwt-bremen.de (D.M.); 3 Institute for Statistics, University of Bremen, Linzer Str. 4, 28359 Bremen, Germany; maendle@uni-bremen.de (A.M.); brannath@uni-bremen.de (W.B.); 4 MAPEX Center for Materials and Processes, University of Bremen, Bibliothekstr. 1, 28359 Bremen, Germany

**Keywords:** high-throughput screening, process chain, heat treatment, particle-oriented peening, optimization

## Abstract

High-throughput screenings are established evaluation methods in the development of functional materials and pharmaceutical active ingredients. The transfer of this approach to the development of structural materials requires extensive adaptations. In addition to the investigation of new test procedures for the determination of material properties and the treatment of metallic materials, the design of experiments is a research focus. Based on given descriptor target values, the statistical design of experiments determines investigations and treatments for the investigation of these materials. In this context, process parameters also have to be determined, as these have a major influence on the later material properties, especially during the treatment of samples. In this article, a method is presented which determines the process parameters iteratively. The validation of the calculated process parameters takes place based on differential scanning calorimetry used as the furnace for the heat treatment of small batches and particle-oriented peening as the characterization method.

## 1. Introduction

For some years, high-throughput screenings have offered the possibility of systematical knowledge gathering with reduced testing effort [1]. In addition to combinatorial approaches, new test strategies significantly reduce the examination time. However, they also demand a systematic design of experiments [2]. Research projects within the field of active substance or functional material development, which are usually binary or tertiary alloys, are frequently using high-throughput methods [3]. They primarily investigate chemical or electrical properties of materials. Contrarily, for metallic structural materials, the mechanical properties are the most important object of investigation. The material properties are influenced by the alloy composition, which contain a large number of elements, and the microstructure of the components [4]. The microstructure results from a combination of alloying processes, as well as from a thermal and mechanical treatment of the components. In summary, compared to binary or tertiary alloys, a more extensive search space is created. 

The materials are evaluated based on empirical descriptors, which can enable conclusions about mechanical material properties [5]. The characterization of a certain material property may involve the determination of several descriptors, in order to make a reliable valuation. Regarding the high-throughput system, this means that a planning step compiles individual combinations of process steps into process chains, depending on the objective of the investigation or the desired material property. These process chains contain a specific combination of individual processes for the generation, treatment, and testing of the samples [6].

The development of experimental design methods requires a special focus on the development of high-throughput systems for structural materials. In [7], given performance profiles describe a target window of the material properties. Within this target window, suitable alloy and treatment combinations are identified. A promising combination is described by the process parameters for the generation of corresponding samples, according to the micro-process function. This includes the specification of suitable parameters for the generation and treatment, as well as the testing of the samples. In the context of the development and evaluation of structural materials, a targeted selection with limited machining and testing capacities is essential. Statistical and heuristic methods are being developed to define areas of interest in order to reasonably limit the search space. By comparison with performance profiles and further investigations, the search areas are concretized. The execution of further experiments and the newly generated data sets refine these search spaces. In this context, the search space describes the target properties at the descriptor or material properties level. The determination of corresponding process parameters for the generation and treatment with which the desired properties are achieved is developed by means of static experimental design procedures [7].

This paper describes an approach to find and evaluate new alloy treatment combinations within a given search space. In this context, a search space describes an area of interest regarding process parameters, which lead to a performance profile approach. The developed method determines process parameters to meet given descriptor values within a defined search space. Using 100Cr6 (AISI 52100) alloy samples, this paper examines the iterative approach of the performance profile using Differential Scanning Calorimetry (DSC) as a small furnace for the heat treatment and particle-oriented peening as a test. With new performance profiles, initial blocks have to be generated by experiments in advance. An iterative process performs model adjusts and initiates new investigations within the search space. This is repeated until alloy and treatment combinations that meet the requirements are found.

## 2. High-Throughput Method and Material

According to [8], high-throughput systems (HTS) were pushed forward, while the pharmaceutical industry was under increasing competitive pressure. Therefore, a need to shorten development cycles while reducing development costs is present. Unlike other industries, development costs for new materials rose by 11% annually [8]. This trend is likely to be stopped by the industrialization of medicine development [9]. Industrialization is particularly characterized by the use of high-throughput systems. These enable a faster acquisition of data from experiments with the lowest possible material consumption, by means of automation, parallelization, and minimization of processes [10]. Accordingly, high-throughput methods are increasingly used in material development. The aim is to increase the experimental throughput, in order to investigate the properties of many different material samples, as well as to optimize them step by step [11]. The development of high-throughput screenings focuses on the design of adapted experiments [12]. These are based on the objective of gaining knowledge about material properties or material behavior in a short time and often with low material input. The knowledge of the entire effect or material behavior is obtained by combining several sequential investigations.

Regarding high-throughput systems of structural materials, this means that the application of standardized test methods is not possible, due to the complex sample geometry, demand of high volumes of material and lengthy investigation procedures. These methods object to HTS, due to their statement accuracy and their duration of the testing. As a result, the high-throughput method ‘Farbige Zustände’ is being developed based on empirical, descriptor-based investigations. Thus, the correlation between these descriptors and the material properties of a material needs to be established. Regarding descriptors, the transfer of gained knowledge is formed by an analytical approach involving a predictor function. Hence, test results can be indirectly compared with material properties [13]. The performance profile can be approximated in an iterative procedure consisting of the process parameters determination and testing.

### 2.1. Method ‘Farbige Zustände’

The method ‘Farbige Zustände’ describes a high-throughput screening approach which investigates material properties of structural materials. The microstructure of the materials is particularly important for the formation of mechanical and chemical properties. For example, depending on the grain size, different strength and ductility is formed. This results in a much more complex search space in HTS compared to high-throughput screening in the pharmaceutical industry and the development of functional materials. The systematic investigation of different alloy and treatment combinations thus requires a high-throughput system in which a large search space can be covered with little material and cost. Springer und Raabe [4] already demonstrated with the rapid alloy prototyping (RAP) method a first approach that can significantly reduce the production and investigation time. To cover the required range of experiments, investigation times have to be further shortened and procedures for the use of micro samples with a diameter of 300 to 1000 µm must be developed in order to save material [6]. In addition to the development of new methods for the generation and treatment of micro samples, organizational approaches are also emphasized in order to accelerate the characterization of materials. Onken mentioned methods with which the throughput time is significantly reduced by the introduction of occupancy planning and logistical control, and productivity is improved by high capacity use [14]. As a further measure to accelerate high-throughput systems, the use of methods for experimental design is described [6]. This determines all process steps that are necessary to achieve an investigation of a material and its microstructure.

**Figure 1:**
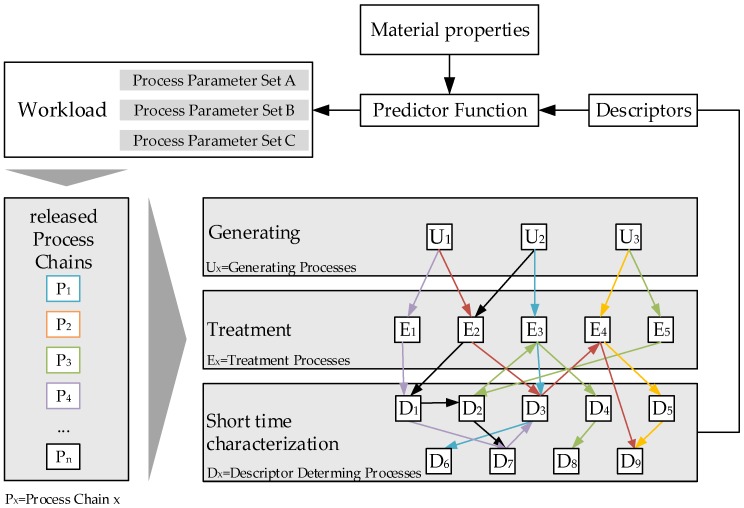
Procedure of the method ‘Farbige Zustände’.

A process parameter set is composed of several components for generation, treatment, and testing (Figure 1). In each process step, a sample-specific parameter definition is carried out based on the given performance profile by a predictor function with given material properties. The alloy composition and the microstructure of the samples are decisive for achievable mechanical properties. These are essential for the process chain-oriented experimental design. Based on the properties that need to be achieved, alloying elements and their mass ratio are determined and supplemented by treatment procedures. Thermal, mechanical, or thermomechanical treatment methods can be used. Thus, the combination of the alloy formation in the sample generation and the selected setup of microstructure processes forms the first stage of the process chain, as shown in Figure 1. In particular, the combination of a thermal and a mechanical process in a sequence can occur in a stepwise generation of the microstructure.

The second part of the process chain consists of a combination of test procedures. This is limited by technical restrictions, due to different requirements of the experiments for embedding and sample preparation [15], as well as due to the addressed material properties in the requirement profile [13].

### 2.2. Material

The experiments were carried out with spherical samples with a diameter of 0.8 mm, which were produced in a roller ball production process of bearing steel SAE52100 (German grade 100Cr6 or AISI 52100). All tested samples were produced within the same batch. However, a slight variation in chemical composition was found in the samples, due to the manufacturing process. The chemical composition of two randomly taken samples measured by optical emission spectroscopy, atomic absorption spectrometry and combustion analysis, as well as the required composition according to DIN EN ISO 683-17:2000-04, are shown in Table 1. The difference between the two samples can mainly be observed due to the nickel content. The chemical composition of the two randomly chosen samples is mainly within the required values. There are only minor exceedances of the DIN limit.

### 2.3. Treatment and Testing of Samples

The microstructure of the samples immediately after the roller ball production consists of martensite with retained austenite and globular carbides. The heat treatments were carried out with a calorimeter of type HT TGA/DSC 3+ (© METTLER TOLEDO, Hamburg, Germany) under an argon atmosphere. The DSC was used to control whether the heat treatment took place below or above the austenitization temperature and due to the high precision of the furnaces and in order to control the transformation points. The samples were heated up to the target temperature with 10 K/min, held at the target temperature for 20 minutes, and cooled to room temperature with 50 K/min. Calibration of the DSC was performed using five different calibration materials (indium, zinc, aluminum, gold, and palladium), which cover the desired temperature range. 

**Figure 2:**
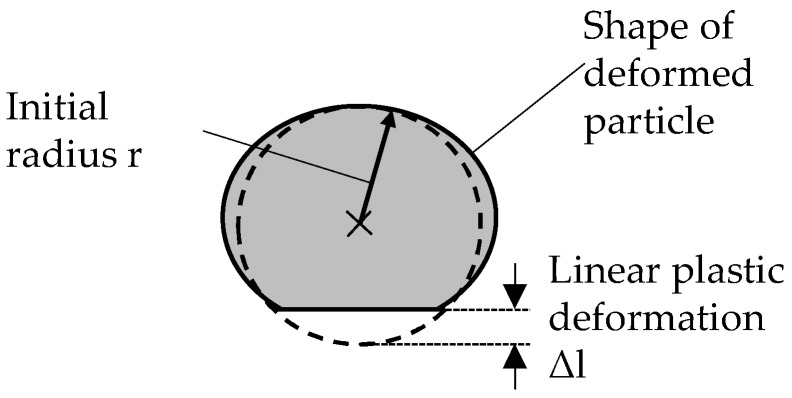
Definition of the determined linear plastic deformation (Adapted from [18].)

The newly established particle-oriented peening setup used in this paper aims at the investigation of mechanical properties of individual particles, after a high velocity impact. This offers the advantage that in contrast to nanoindentation [17], no polished surface is required and greater deformation takes place, which is more robust against micro-influences. It allows for quick deformation of a large number of particles in a highly defined and reproducible way. For this purpose, each micro sample is accelerated with compressed air (max. jet pressure p_s_ 4 bar ≙ max. particle velocity of approx. 70 m/s for the investigated material) to impact on a hardened contact plate of 100Cr6 (60 HRC), which is located at a constant distance of 80 mm in front of the nozzle outlet. This avoids plastic deformation of the contact plate, while the particles are deformed by the impact according to their material properties (e.g., hardness or yield strength). Therefore the observed plastic deformation allows conclusions to be drawn about the mechanical properties of the samples. The particles, as well as the plastic deformation resulting from the impact, are analyzed using the Zeiss SteREO.V12 microscope in combination with the REOObjective.435200-0000-000 objective. The descriptor ‘linear plastic deformation’ is determined after the impact. It is defined as the difference between the initial particle radius r and the distance from the center to the flattened surface after the impact (see. Figure 2). The relationship between the determined plastic deformation and material properties has already been demonstrated in Kämmler et al [18]. For rather distinctive material properties (X210Cr12 vs. AlSi12), it was shown that different hardness values result in different descriptor values. As shown in [19], significantly differing DSC heat treatments can be characterized using the particle-oriented peening process. It is, therefore, assumed that a more fine-grained variation of the heat treatment parameters, as conceivable in the context of current investigations, will lead to distinguishable descriptor values.

### 2.4. Design of Experiments and Routing of Processchains

Combinatorial material analysis places a different requirement on the design and implementation of process chains than production [14]. By definition, a parameter window, which needs to be as wide as possible, should be covered by the tests. This is reflected in a high number of variants. Depending on the desired resolution and the statistical significance of the investigations, such a system occupies an intermediate position between individual production (one sample per possible combination) and individualized mass production (extensive random samples per variant). However, a completely synchronized production line cannot be realized under these requirements, as it differs fundamentally in its structure. In contrast to the usually existing linear and fixed connection of all sub-processes and investigations, the connection is flexible during material testing. Schneider et al. [20] describe an undirected, recursive material flow, which is caused by repetitive treatments and testing. In particular, the authors point to the strong effect of rejected samples, which leads to a significant change of the investigation effort as the process chain progresses [20].

## 3. Evaluation and Discussion of Descriptors

The repeated execution of examinations, as well as the setup of the microstructure and the multiple processing of a process chain altogether leads to a significant additional expenditures, within the high-throughput system. Thus, the quality of the iterative predictions of process parameters, which ought to determine a suitable combination for achieving the required material properties, has a high influence on the outcome of the investigation. 

**Figure 3:**
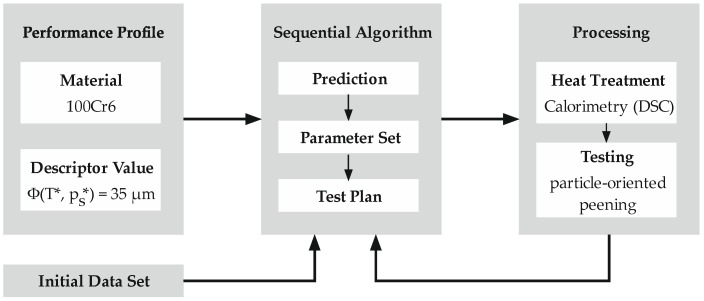
Procedure for the iterative determination of process parameters.

In this approach, the adaptation of the process parameters of the thermal treatment is available for the determination of a requirement profile. Varying heat treatment has a particular influence on the microstructure adjustment and thus, on the mechanical properties of the sample. The heat treatment is carried out by means of the DSC, while the peening process functions as a method for determining the sample properties. The process parameters used in the particle-oriented peening process are also determined within the sequential algorithm. This sequential sample data-based approach (Figure 3) is presented to find process parameters, which produce a desired descriptor value.

### 3.1. Data

**Figure 4:**
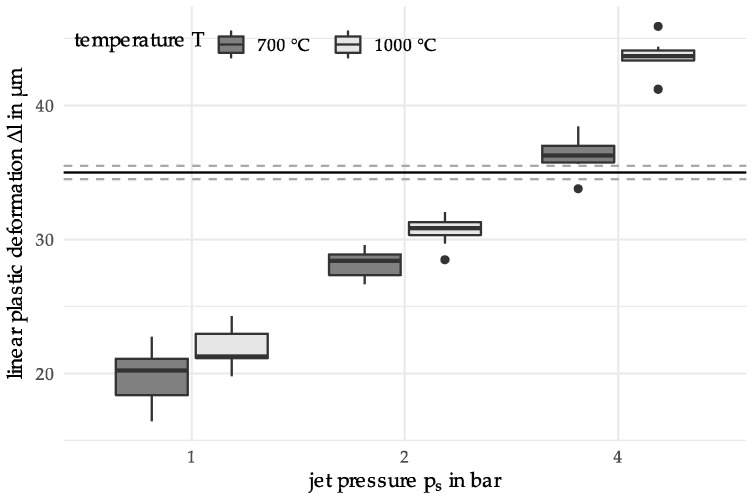
Boxplot for the linear plastic deformation of the initial sample data.

The initial data set consists of material samples whose properties (=output parameters) are determined by variation of the two input parameters, the heating temperature T (700 °C and 1000 °C) and the jet pressure $p_{s}$ (1 bar, 2 bar and 4 bar). These temperature parameters were chosen to generate results that were DSC heat-treated with a heating temperature below and above the austenitization temperature of 100Cr6 (approx. 850 °C). The temperature was limited to 1100 °C because the carbides dissolve above. The selection of the jet pressures is based on the experimentally possible range. For each of the 2×3 parameter combinations, 10 samples were produced, i.e., for the initially 6 different materials 10 repeated measurements of the output parameter, linear plastic deformation Δl, were performed. Another name for the input parameters is predictors, while the output parameter is also known as the descriptor. The initial data set is depicted in Figure 4, as follows.

The horizontal lines around Δl=35 μm depict a later defined target value with a surrounding target region. The boxplot meets the expectations that the variability of the measurements diminishes for increasing jet pressure, while having a temperature of 700 °C higher than for 1000 °C.

We assume there is an unknown functional relationship between the heating temperature and jet pressure as explaining variables and linear plastic deformation as explained variable, i.e.:

(1) $$\Delta l=\Phi \left(T,p_{s}\right)$$

We denote the i-th observation of the explaining variables T and $p_{s}$ of the sample data as bivariate predictor variable $p^{\left(i\right)}=\left(p_{T}^{\left(i\right)},p_{ps}^{\left(i\right)}\right)\in {\mathbb{R}}^{2}$, where the lower indices T and ps identify parameters for temperature and jet pressure respectively. The upper index (i) here and in the following is the sample ID, where i∈1,…,n for n measurements. The i-th observation of the explained variable Δl of the sample data set is denoted as descriptor $d^{\left(i\right)}\in \mathbb{R}$. Due to inaccuracies in the measurements and the tuning of the process parameters the unknown functional relationship is assumed to hold for the samples up to an error term $\varepsilon^{\left(i\right)}$ with expectation $E\left[\varepsilon^{\left(i\right)}\right]=0$, i.e.:

(2) $$d^{\left(i\right)}=\Phi \left(p_{T}^{\left(i\right)},p_{\mathrm{ps}}^{\left(i\right)}\right)+\varepsilon^{\left(i\right)}$$

### 3.2. Optimization Problem

We assume that a process is required, which produces a material sample with a certain linear plastic deformation as target value, e.g., ${\Delta l}^{*}=35\;$µm. The aim is then to find values $T^{*}$ and $p_{s}^{*}$ for heating temperature and jet pressure such that $\Phi \left(T^{*},p_{s}^{*}\right)=35\;$µm.

### 3.3. Method

As the optimization problem is a single objective optimization problem with an only 2-dimensional decision space, a great number of available standard approaches for optimization could be applied. As an example of a posteriori method which delivers the experimenter a whole set of possible Pareto optimal solutions the approaches NSGA-II [21], and SMS-EMOA [22] shall be mentioned here, as there are implementations of these algorithms in optional packages for the statistical software R. 

**Figure 5:**
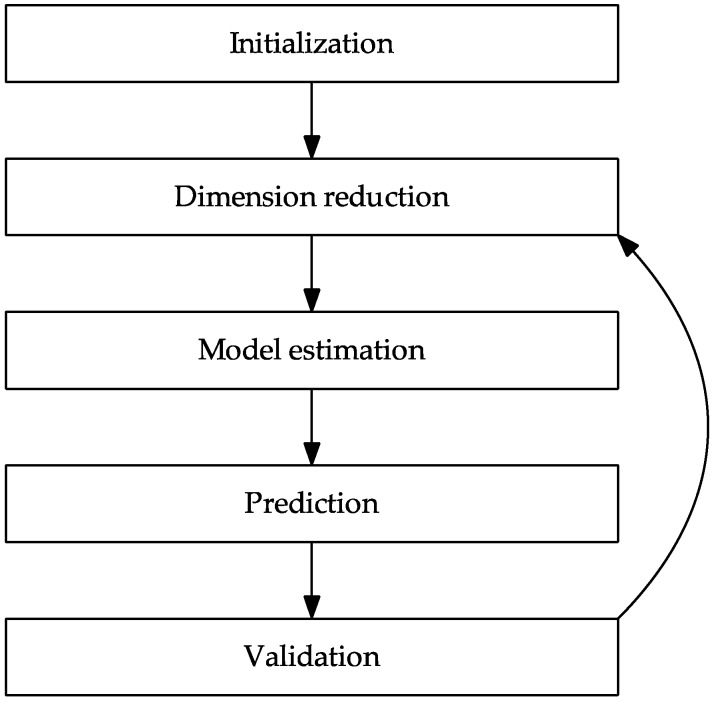
Steps of the sequential algorithm.

The aim of this work is to determine at least one single solution for the predictors, which has a corresponding linear plastic deformation. Process parameter combinations with corresponding descriptor values in a previously determined target region around the target value ${\Delta l}^{*}$ are considered to be solutions. Based on dimension reduction, an alternative approach for this optimization problem is suggested for the case of a two dimensional parameter space. In contrast to the previously mentioned multi criteria optimization algorithms, it refrains from determining a whole Pareto front of Pareto optimal solutions and concentrates on returning a desirable solution as fast as possible. In this context, *fast* means requiring only a relatively small number of samples. The following Figure 5 depicts the general steps.

The target region for the desired descriptor value in µm is chosen as $\Delta_{l}^{*}\Delta \left[35-\delta ,35+\delta \right],\;$δ=1. The search space is limited to T, 400≤T≤1100 and $p_{s}$, $1\leq p_{s}\leq 4$. These limits are based on material knowledge as well as measurement and process-related limitations. Since the examined particles are martensitic in their initial state, it can be assumed that below 200 °C only very slight changes in hardness can be observed. At a temperature of 400 °C, a significant reduction in hardness can be expected. Temperatures above 1100 °C are not considered due to carbide dissolution [17]. As previous investigations showed, plastic deformations for jet pressures below 0.7 bar can no longer or only with difficulty be determined [18], thus the minimum jet pressure of the current investigation was set at 1 bar. The upper limit for the jet pressure results from the current experimental setup where only a maximum jet pressure of 4 bar can be realized.

Initial measurements were performed as depicted above in Figure 4. The parameter variations of T and $p_{s}$ were chosen by the experimenter with respect to the following two objectives: First, the predictor parameters shall be chosen in such a way that the expected observations depend heavily on the chosen predictor values. This will ensure that the data contain information on the effect of the parameter variation. Second, the chosen parameter values shall enclose the area of interest, where we expect the desired solution will be located. It is preferable that the desired solution is inside this specified area, because predictions from an interpolation are generally more stable than extrapolations. 

The number of iterations of the algorithm will be restricted to 2 in this data example. It is obvious that in practice usually more iterations will be needed. Here, two iterations shall suffice to present the framework of our approach. Whereas in theory iterations would be continued until a solution for the optimization problem with sufficient accuracy is found, in practice it is a given time or cost limit which additionally limits the maximum number of iterations.

After the initialization procedure, dimension reduction approaches will be applied. The descriptor $d^{\left(i\right)}$ is already univariate, i.e., no dimension reduction has to be performed on the descriptor space. Otherwise, a principal component analysis would were performed for the descriptor.

The variables of the 2-dimensional predictor space will be transformed using the PLS1 algorithm [23]. PLS stands for “Partial Least Squares” or “Projection to Latent Structures” and is an approach, which is used for dimension reduction in, usually linear, multivariate regression models. Within the applications presented in this paper, the PLS1-implementation in the function *plsreg1* of the R-package *plsdepot* is used. This PLS-based dimension reduction transforms the problem of finding solutions for

(3) $$\Phi \left(p_{T}^{\left(i\right)},p_{ps}^{\left(i\right)}\right)=E\left[d^{\left(i\right)}|p_{T}^{\left(i\right)},p_{ps}^{\left(i\right)}\right]$$

to the problem of finding solutions for

(4) $$\tilde{\Phi }\left(x^{\left(i\right)}\right)=E\left[d^{\left(i\right)}|x^{\left(i\right)}\right],$$

where the $x^{\left(i\right)}=\left(x_{1}^{\left(i\right)},x_{2}^{\left(i\right)}\right)$ are orthogonal linear transformations of the $p_{T}^{\left(i\right)},p_{ps}^{\left(i\right)}$ to a new coordinate system. We call $x^{\left(i\right)}$ the pseudo predictor. The transformation according to the PLS1 algorithm maximizes the covariance between the first pseudo predictor $x_{1}^{\left(i\right)}$ and the descriptor $d^{\left(i\right)}$.

Assuming that the dependence between predictors and descriptors is mainly determined by $x_{1}^{\left(i\right)}$, the second pseudo predictor $x_{2}^{\left(i\right)}$ can be neglected. In this paper, this simplifying assumption is made: Thus, a dimension reduction to a 1-dimensional predictor is performed and a 1-dimensional relationship

(5) $$f\left(x_{1}^{\left(i\right)}\right)=E\left[d^{\left(i\right)}|x_{1}^{\left(i\right)}\right]\left(\approx E\left[d^{\left(i\right)}|x^{\left(i\right)}\right]\right).$$

between the first component of the pseudo predictor and the descriptor is assumed.

In the now following modelling step, this unknown function f(⋅) will be modeled as polynomial function of order k, 1≤k≤5, using weighted least squares regression (WLS-regression). Polynomials of higher orders were not permitted to prevent overfitting.

The choice of the actual polynomial order k is done based on the BIC (Bayesian Information Criterion): The BIC is a criterion for model selection among a finite set of models. It is implemented as a function for linear models in common statistics software and mathematically defined as

(6) $$\mathrm{BIC}=\ln\left(n\right)k-2\ln\left(\hat{L}\right),$$

with $\hat{L}$ being the maximized value of the likelihood function of the fitted model, n the number of observations and k the number of parameters estimated by the model. 

For each order 1, …, 5 the corresponding model is determined as described below and then the BIC is calculated. Finally, the polynomial order of the model k ∈ {1,…,5} will be chosen such that the resulting model leads to the lowest value of the BIC.

**Figure 6:**
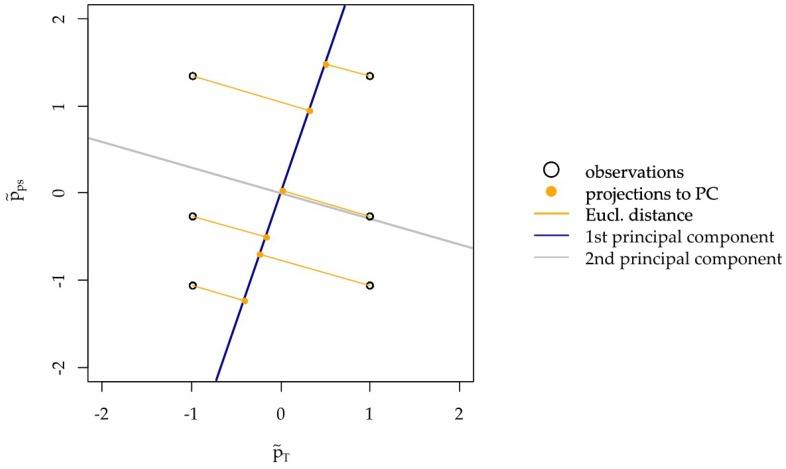
Normalized predictors and their distance to their 1-dimensional projections.

Now the weighted regression is performed. A weighted regression approach is preferred instead of usual non-weighted least squares regression to account for the distance between the original observations and their dimensionally reduced counterparts. This way pseudo predictor values, which represent the original predictor values better will have a stronger influence in the regression. In the following graph denote the normalized predictors as ${\tilde{p}}_{}^{\left(i\right)}=\left({\tilde{p}}_{T}^{\left(i\right)},{\tilde{p}}_{\mathrm{ps}}^{\left(i\right)}\right)$ with coordinates ${\tilde{p}}_{T}^{\left(i\right)}\Delta \frac{p_{T}^{\left(i\right)}-{\hat{\mu }}_{T}}{{\hat{\sigma }}_{T}},\;{\tilde{p}}_{\mathrm{ps}}^{\left(i\right)}\Delta \frac{p_{\mathrm{ps}}^{\left(i\right)}-{\hat{\mu }}_{\mathrm{ps}}}{{\hat{\sigma }}_{\mathrm{ps}}}$ for i=1,…,n, where ${\hat{\sigma }}_{i}$ and ${\hat{\mu }}_{i}$, i∈{T,ps} are the empirical variance and mean of the predictor observations for temperature and jet pressure. The regression weights $w_{i}$ are chosen based on the squared Euclidean distance between the normalized predictor observations and their projections onto the first principal component from the previously performed PLS1. More specifically the weights are defined by $w_{i}={\left(x_{2}^{\left(i\right)}\right)}^{2}$ (see Figure 6).

The weighted least squares coefficients $\beta =(\beta_{0},\ldots ,\beta_{k})$ for the polynomial regression will be estimated by

(7) $$\mathrm{WLS}\left(\beta \right)=\arg\underset{\beta }{\min}\overset{n}{\underset{i=1}{\sum }}\frac{1}{w_{i}}{\left|d_{}^{\left(i\right)}-\overset{k}{\underset{j=0}{\sum }}{\left(x_{1}^{\left(i\right)}\right)}^{j}\beta_{j}\right|}^{2}$$

As a result, one gets the regression model

(8) $${\hat{f}}_{1}\left(x_{1}\right)=\overset{k}{\underset{j=0}{\sum }}\beta_{j}{\left(x_{1}\right)}^{j}=\Delta_{l}.$$

Now candidates for appropriate pseudo predictors will be determined as roots of

(9) $${\hat{f}}_{1}\left(x_{1}^{*}\right)=\overset{k}{\underset{j=0}{\sum }}\beta_{j}{\left(x_{1}^{*}\right)}^{j}=\Delta_{l}^{*}.$$

Each of its roots can be regarded as a solution to the simplified optimization problem in the 1-dimensional pseudo descriptor space. For practical use and interpretability, its coordinate has to be transformed into the original 2-dimensional space, consisting of values for temperature and jet pressure. Let ${\hat{x}}_{1}^{*}$ denote one of the estimated roots. It will be shown in the following how the transformation to the original parameter space is performed. Denote the predictor variables as $P=\;{\left(p^{\left(1\right)},\ldots ,p^{\left(n\right)}\right)}^{T}$, the corresponding scores (pseudo predictors) as $X={\left(x^{\left(1\right)},\;\ldots ,x^{\left(n\right)}\right)}^{T}$ and V the matrix which suffices

(10) P=XV.

In common implementations of PLS1, the matrix V is returned together with the scores X; in the R package *plsdepot* V is called the matrix of the modified weights. Denote by $V_{1,\cdot }$ the first row of V. Then, the coordinates of the pseudo predictor ${\hat{x}}_{1}^{*}$ in the standardized predictor space can be determined as z,

(11) $$z^{T}:=\left({\hat{x}}_{1}^{*}\right)\cdot V_{1,\cdot }$$

The coordinates in the original predictor space are then ${\hat{p}}^{*}=\;{\left({\hat{p}}_{T}^{*},{\hat{p}}_{ps}^{*}\right)}^{T}$, with ${\hat{p}}_{i}^{*}=z_{i}\cdot {\hat{\sigma }}_{i}+{\hat{\mu }}_{i},\;i\in \left\{T,p_{s}\right\}$, with ${\hat{\sigma }}_{i}$ and ${\hat{\mu }}_{i}$ as above.

It is possible that the above prediction of possible solutions fails. If the above Equation (3) cannot be solved by any $x_{1}^{*}$ this does not necessarily mean that no solution of a predictor with its corresponding descriptor being located in the target region exists. Therefore, a fallback procedure is required to expand the current sample of observations. This may happen according to the decision of the experimenter who can suggest interesting areas, from which new samples could improve the behavior of the algorithm. Alternatively, if an automated solution is necessary, the point with maximal distance to its closest observation in the (normalized) search space can be determined and taken as new prediction. This will stepwise fill up the gaps in the process parameter space. 

Finally, the validation of the new solutions has to be performed. This paper will be restricted to assessing the quality of the result using boxplots as an exploratory tool. The approach can obviously be expanded by introducing a formal stopping rule for repeated measurements in the validation step, if necessary. A sample size estimation can give additional advice on how many samples might be needed to assess the quality of the solution based on quantitative arguments. 

### 3.4. Results

Based on the initial measurements, a new set of parameter values for new repeated measurements was suggested for temperature T=880 °C  and jet pressure $p_{s}=3.06\;\mathrm{bar}$, see the corresponding boxplot in the following graph. Although for the predicted location there is a measurement with deformation larger than the target value, the boxplot generally suggests that the mean deformation for the predicted process parameters is lower than 35 µm; even the upper quartile is located at deformation value 34 µm. Assuming the error for the measurements is normally distributed we get as 95% confidence interval (30.5, 34.2). 

**Figure 7:**
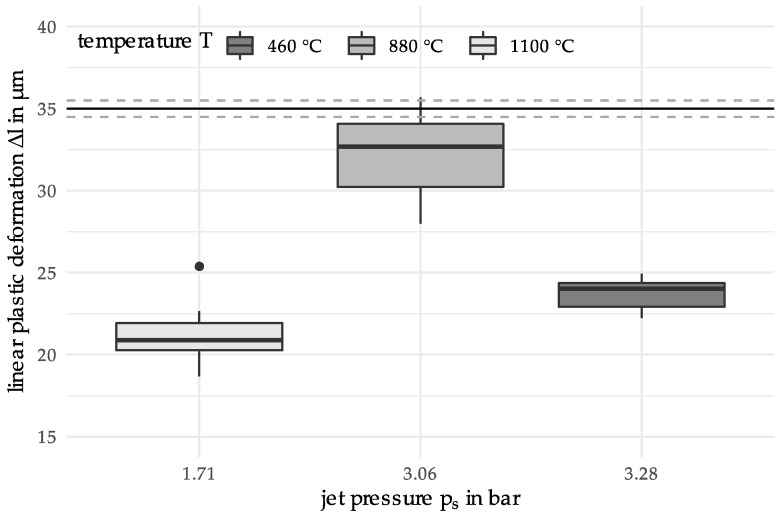
Boxplots for the new measurements; second boxplot corresponds to the suggested predictor values in iteration 1.

**Figure 8:**
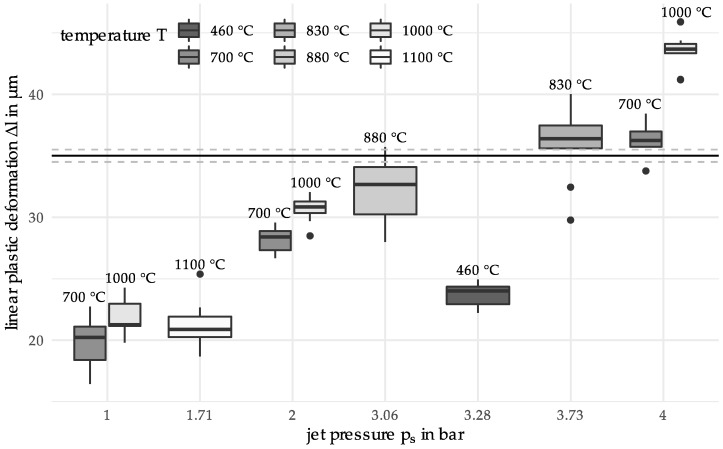
Boxplot comparison of the data of all measurements.

Further measurements at (T=1100 °C, $p_{s}=1.71\;\mathrm{bar}$) and (T=460 °C, $p_{s}=3.28\;\mathrm{bar}$) were performed (see Figure 7). These values were not directly suggested by the algorithm, but they were chosen by the experimenter to widen the search space. The idea of the evaluation of these two extra points was first to get measurements for higher and lower temperature levels. These values were chosen as points on the second principal component with temperature values 460 °C and 1100 °C. The scale and location of the repeated measurements is summed up graphically in the following boxplot:

In the next iteration the algorithm suggests process parameters $\left(T=830\;{}^{\circ}C,\;\;p_{s}=3.73\;\mathrm{bar}\right)$. A table of all input and output parameters can be found in the 03278ix A. In the following graph the resulting boxplots of all so far measured parameter sets are shown together (see Figure 8). For $\left(T=830\;{}^{\circ}C,\;p_{s}=3.73\;\mathrm{bar}\right)$ the boxplot suggests generally a too high linear plastic deformation is achieved; however, two observations which are possible outliers are much lower with deformation values of 29.8 µm and 32.5 µm.

For ten measurements, we get the 95% confidence interval (33.8, 37.9). We have an interval width of 4.1, i.e., a margin of error of 2.05. 

Considering the objective to find process parameters which have a mean deformation value in the target region $\Delta_{l}\;:=\left[35-\delta ,35+\delta \right],\;\;\delta =1$, it has to be accepted that the number of measurements is too low for data with the observed standard deviation of 2.79 for the finally suggested predictor values. To illustrate this, consider a sample size estimation is performed under the assumption of normal errors and assuming the empirical standard deviation as the real underlying standard deviation. Then, assuming the standard deviation 2.79, several n=33 measurements is needed in order to achieve a margin of error smaller than 1. 

In addition to the mathematical analysis, the consideration of the experimental set-up as well as a material science-based interpretation of the results are reasonable. Starting with the initial measurements (see Figure 3; T = 700 °C, 1000 °C; p_s_ = 1 bar, 2 bar, 4 bar), the statistical dispersion of the linear plastic deformation decreases with increasing jet pressure. This is due to the fact that comparatively small plastic deformations occur at a jet pressure of 1 bar. Although these can be measured, there is a higher uncertainty. Furthermore, a higher dispersion can be observed for 700 °C than for 1000 °C. At a heating temperature of 1000 °C, the austenitization temperature is already exceeded. However, this temperature, in combination with the holding time, is not sufficient for a complete phase transformation. Thus, a mixed microstructure with ferrite and a small amount of perlite still results. Lower heating temperatures such as 700 °C mainly result in tempering of the previously martensitic microstructure. This reduces the hardness compared to the initial state. Since a higher hardness results in a greater resistance to deformation, the determined linear plastic deformation has its lowest values for material states of high hardness. The higher plastic deformation determined at 1000 °C can be explained by the fact that when tempering at 700 °C there is still remaining martensite, which has a higher hardness than the microstructure resulting from a heating temperature of 1000 °C. Considering the results of the two iterations, an annealed martensite structure can also be assumed for the heating temperatures 460 °C, 830 °C and 880 °C. While the boxplots for 460 °C and 830 °C depict interquartile ranges comparable to 700 °C, the range for 880 °C is much larger. As observed in [19] this may be explained by a decarburization which has occurred near the surface of the particles. Since the temperature resulted in the material being in a two-phase region for the duration of the holding time, a mixed structure of ferrite, perlite, upper bainite and carbides was created. Since the particle-oriented peening mainly causes a deformation of the particle surface and subsurface area, the observed microstructural effects in the outer surface and subsurface layers (decarbonized) may affect the determined plastic deformation. Compared to [19] the jet pressure was lower during all the experiments in this paper. The lower pressure results in a smaller deformation and a higher relative error in the measurements. This explains the higher dispersion of the results. Based on the results of Toenjes et al. [19] it can further be assumed that a complete austenitization occurred at the temperature of 1100 °C. Therefore, a new microstructure was formed, resulting in a higher hardness and thus also a lower plastic deformation [19]. The results shown in Figure 8 can be attributed to the microstructure, which is caused by the DSC heat treatment. To ensure lower deviations of the descriptor values when considering the complete temperature range (from 400 °C to 1100 °C) an optimization of the problem should therefore be carried out on a sectoral basis. Materials knowledge could, for example, be used to redefine the limits of the search space.

## 4. Conclusions

The paper presents a coherent system and structure for high-throughput systems for structural materials. The strong link between process chain design, logistics, experimental design, and statistics enables a new approach. In this paper, it is pointed out that the statistical approach is essential for the design of experiments within high-throughput systems. The procedure has a great impact of the scheduling of process chains and operating steps. In conjunction with the precision and number of iterations, the testing requires various resources regarding type and quantity of treatment and testing processes.

The paper presents a statistical procedure for a descriptor-based parameter optimization in high-throughput systems for structural materials. The presented approach relies on the assumption that the relationship between predictors and descriptors can be described by the relationship between a dimensionally reduced 1-dimensional pseudo predictor and pseudo descriptor. If the simplifying assumption cannot be justified, a refined algorithm, which models more than one principal component, should be used. The validation of the method includes the DSC to set up the microstructure, as well as the particle-oriented peening to test the samples’ properties. The paper shows that it is possible to determine process parameters which produce a descriptor that approximates the previously defined target values within two iteration steps. To widen the field of application for the presented method, variations of boundary conditions can be considered. For this purpose a special treatment of discrete transformation points of the material can be included in the mathematical approach. For the examined material (100Cr6), a transformation occurs for example when the temperature exceeds 850 °C. 

In the high-throughput, several iterations of alloys are investigated parallelly. For the application of the method within the experimental design, it may be useful to prioritize individual investigations. This way, experiments for the exploration of new search areas can be accelerated. In addition, it is possible to supplement the termination criteria of the iteration with a consideration of the informative value for the evaluation of a new material. In particular, the fundamental conflict between a good system use and the benefit of the experiments in terms of content have to be weighed up in this context.

## Figures and Tables

**Table 1:** Chemical composition of the used alloy SAE52100 in wt.% in comparison with the required chemical composition of the DIN EN ISO 683-17:2000-04.

Material		Chemical Composition in wt.%							
		Fe	C	Cr	Mn	Ni	P	S	Si
Samples Test 1 ^b^	-	bal.	1.03 ^c^	1.20	0.38	0.40		0.015 ^c^	0.35 ^a^
Samples Test 2 ^b^	-	bal.	1.07 ^c^	1.31	0.35	0.17		0.018 ^c^	0.35 ^a^
DIN EN ISO 683-17:2000-04 [16]	min max	bal.	0.93 1.05	1.35 1.60	0.25 0.45	0.00 0.40	- 0.025	- 0.015	0.15 0.35

^a^ by optical emission spectroscopy (OES), ^b^ by atomic absorption spectrometry (AAS), ^c^ combustion analysis.

## Abbreviations

${}^{\left(i\right)}$	upper indices in parentheses contain the sample ID, here i
${}^{T}$	transpose of a matrix or vector
β	vector of coefficients from weighted least squares (WLS)-regression in Equation (7), $\beta =\left(\beta_{1},\;\ldots ,\beta_{k}\right)$
$d^{\left(i\right)}$	i-th observation of descriptor, see Equation (2)
Φ	real relationship between predictors and descriptors, see Equation (1)
$\tilde{\Phi }$	real relationship between pseudo predictors and pseudo descriptors, see Equation (4)
Δl	linear plastic deformation [µm]
$\Delta l^{*}$	target value for linear plastic deformation; equals 35 μm, see page 7
δ	half width of the target region for the linear plastic deformation
E[⋅]	expectation
E[⋅|⋅]	conditional expectation
$\varepsilon^{\left(i\right)}$	model error term in Equation (2)
f	functional relationship between the first pseudo predictor and pseudo descriptor, see Equation (5)
${\hat{f}}_{1}$	regression model for $x_{1}$ and $\Delta_{l}$(8)
k	polynomial order in the model estimation step, see page 9
${\hat{\mu }}_{T}\;,{\hat{\mu }}_{ps}$	empirical mean of T, $p_{s}$
n	number of joint observations of predictors and descriptors
P	matrix of predictors, see Equation (10)
$p^{\left(i\right)}$	vector of predictors of the i-th observation, see page 7
$p_{ps}^{\left(i\right)}$	jet pressure of i-th observation, see page 7
$p_{s}$	jet pressure [bar]
$p_{s}^{*}$	optimal jet pressure
$p_{T}^{\left(i\right)}$	temperature of i-th observation, see page 7
${\tilde{p}}_{}^{\left(i\right)}$	normalized predictors ${\tilde{p}}_{}^{\left(i\right)}=\left({\tilde{p}}_{T}^{\left(i\right)},{\tilde{p}}_{ps}^{\left(i\right)}\right)$, see page 9
${\tilde{p}}_{ps}^{\left(i\right)}$	second coordinate of ${\tilde{p}}_{}^{\left(i\right)}=\left({\tilde{p}}_{T}^{\left(i\right)},{\tilde{p}}_{ps}^{\left(i\right)}\right)$
${\tilde{p}}_{T}^{\left(i\right)}$	First coordinate of ${\tilde{p}}_{}^{\left(i\right)}=\left({\tilde{p}}_{T}^{\left(i\right)},{\tilde{p}}_{ps}^{\left(i\right)}\right)$
${\hat{p}}^{*}$	transformation of ${\hat{x}}_{1}^{*}$ to the standardized predictor space of temperature and jet pressure, see Equation (11)
${\hat{p}}_{ps}^{*}$	jet pressure corresponding to ${\hat{x}}_{1}^{*}$ in the standardized predictor space, see page 10
${\hat{p}}_{T}^{*}$	temperature corresponding to ${\hat{x}}_{1}^{*}$ in the standardized predictor space, see page 10
r	radius of the particle [mm]
${\hat{\sigma }}_{T}$, ${\hat{\sigma }}_{ps}$	empirical standard deviations of T, $p_{s}$
T	temperature [°C, K]
$T^{*}$	optimal temperature
V	matrix of modified weights, defined by Equation (10)
$V_{1,\cdot }$	first row of matrix V
X	matrix of scores, see Equation (10)
$x^{\left(i\right)}$	pseudo predictor of i-th observation, see Equation (4)
${\hat{x}}_{1}^{*}$	estimator for optimal pseudo predictor coordinate, see Equation (9)
$w_{i}$	weight for i-th observation in WLS-regression in Equation (7)
wt%	percentage by weight
z	transformation of ${\hat{x}}_{1}^{*}$ to the standardized predictor space, see Equation (11)

## Appendix A

**Table A1:** Input and output parameters for all iterations.

Iteration	Temperature in °C	Pressure in bar	Iteration	Temperature in °C	Pressure in bar
0	1000	4	0	700	1
0	1000	4	0	700	1
0	1000	4	0	700	1
0	1000	4	0	700	1
0	1000	4	0	700	1
0	1000	4	0	700	1
0	1000	4	0	700	1
0	1000	4	0	700	1
0	1000	4	1	1100	1.71
0	1000	4	1	1100	1.71
0	1000	2	1	1100	1.71
0	1000	2	1	1100	1.71
0	1000	2	1	1100	1.71
0	1000	2	1	1100	1.71
0	1000	2	1	1100	1.71
0	1000	2	1	1100	1.71
0	1000	2	1	1100	1.71
0	1000	2	1	1100	1.71
0	1000	2	1	880	3.06
0	1000	2	1	880	3.06
0	1000	1	1	880	3.06
0	1000	1	1	880	3.06
0	1000	1	1	880	3.06
0	1000	1	1	880	3.06
0	1000	1	1	880	3.06
0	1000	1	1	880	3.06
0	1000	1	1	880	3.06
0	1000	1	1	880	3.06
0	1000	1	1	460	3.28
0	700	4	1	460	3.28
0	700	4	1	460	3.28
0	700	4	1	460	3.28
0	700	4	1	460	3.28
0	700	4	1	460	3.28
0	700	4	1	460	3.28
0	700	4	1	460	3.28
0	700	4	1	460	3.28
0	700	4	1	460	3.28
0	700	2	2	830	3.73
0	700	2	2	830	3.73
0	700	2	2	830	3.73
0	700	2	2	830	3.73
0	700	2	2	830	3.73
0	700	2	2	830	3.73
0	700	2	2	830	3.73
0	700	2	2	830	3.73
0	700	2	2	830	3.73
0	700	2	2	830	3.73
0	700	1	2	830	3.73
0	700	1			
